# Four-Year Outcome of Aflibercept for Neovascular Age-Related Macular Degeneration and polypoidal choroidal vasculopathy

**DOI:** 10.1038/s41598-019-39995-5

**Published:** 2019-03-06

**Authors:** Keiichi Nishikawa, Akio Oishi, Masayuki Hata, Masahiro Miyake, Sotaro Ooto, Kenji Yamashiro, Manabu Miyata, Hiroshi Tamura, Naoko Ueda-Arakawa, Ayako Takahashi, Yu Kawashima, Akitaka Tsujikawa

**Affiliations:** Department of Ophthalmology and Visual Sciences, Kyoto University Graduate School of Medicine, Kyoto, Japan

## Abstract

Intravitreal injections of anti-vascular endothelial growth factor agents such as ranibizumab and aflibercept are the first-line treatment for neovascular age-related macular degeneration (AMD). However, data about long-term outcome in real-world clinical practice is scarce. We recruited 98 AMD patients and investigated four-year visual outcome. During the four years, 25 patients dropped out. The survivors received 7.0 ± 0.1 injections during the first year and 8.0 ± 7.4 injections in the following three years. The logarithm of minimum angle of resolution (logMAR) at baseline, year one, and year four was 0.28, 0.14 (P = 0.033), and 0.22 (P = 0.697), respectively. The gain of vision was not different among AMD subtypes (typical AMD, polypoidal choroidal vasculopathy, and retinal angiomatous proliferation; P = 0.513) Among the investigated factors, the presence of external limiting membrane (ELM), the absence of vitreoretinal adhesion, and thicker choroid at baseline were associated with better logMAR values at year four (coefficient beta = −0.388, 0.201, and −0.001; P = 7.34 × 10^−6^; 0.01, and 0.028, respectively). In the present study, vision was retained at baseline level after the four-year treatment with aflibercept. The status of ELM, vitreoretinal adhesion, and choroidal thickness were predictive factors for final vision.

## Introduction

Age-related macular degeneration (AMD) is a leading cause of blindness in developed countries^1^. Late-stage AMD is characterized by geographic atrophy and choroidal neovascularization (CNV). While geographic atrophy progresses gradually, CNV causes hemorrhage and exudative changes in the macula, leading to rapid vision loss. Indeed, CNV is responsible for more than 80 percent of severe visual loss or legal blindness in patients with AMD^2^.

While there is no established cure for geographic atrophy, CNV can be controlled with vascular endothelial growth factor (VEGF) inhibitors. The anti-VEGF agents inhibit CNV growth and fluid leakage, which enable visual improvement. However, the treatment has some drawbacks, including a need for frequent intravitreal injections; additionally, it is not easy to strictly continue this regimen in clinical practice. Thus, visual gain in the first year is generally favorable, but long-term outcome is not as promising. For example, HORIZON and a subsequent SEVEN-UP study showed that visual acuity improved during monthly ranibizumab treatment and gradually declined thereafter^3,4^. Another large clinical trial, CATT, reported five-year follow-up data showing similar results; after the release from the trial protocol, visual acuity dropped to baseline level^5^.

While the above-mentioned studies used ranibizumab or bevacizumab, there are other treatment options available, such as pegaptanib and aflibercept. Particularly, aflibercept is potentially more effective because it also blocks placental growth factor, has a higher affinity for VEGF, and has a longer half-life^6^. In fact, two-monthly injections of aflibercept achieved visual gain identical to monthly ranibizumab^7,8^. In addition, switching to aflibercept in ranibizumab-refractory cases generally shows anatomical improvement^9,10^. Thus, we predicted that aflibercept may yield better long-term visual outcomes than ranibizumab.

When considering long-term visual outcome, identifying predictive factors for good or poor vision is important. Because it is not easy to continue intensive treatment for all patients, identifying those at high-risk and investing resources for these patients would improve the visual outcome of the overall cohort.

In the present study, we investigated the four-year visual outcome of aflibercept treatment in patients with AMD and explored the factors associated with final vision.

## Methods

### Study design

Prospective, non-randomized, interventional study.

The study design was approved by the Institutional Review Board of Kyoto University Graduate School of Medicine, and all study conduct adhered to the tenets of the Declaration of Helsinki. Each patient gave written informed consent for the participation in the study.

### Patients and study population

We included treatment-naïve AMD patients between December 2012 and December 2013 at Kyoto University Hospital. We had already reported one-year results^11^, causes of vision loss^12^, and recurrence rate in the second year^13^. Thus, this is a consecutive study based on the previous research.

The inclusion criteria included an age older than 50 years, axial length less than 26.5 mm, the presence of neovascular AMD, and the willingness to participate in the study. The exclusion criteria included any previous treatment of CNV and the presence of other retinal diseases such as angioid streaks, vitelliform macular dystrophy, and retinal vein or artery occlusion. Those with chronic courses of AMD, indicated by disease history and/or massive fibrotic lesions, were also excluded.

### Intervention and observation procedure

Each participant underwent three monthly injections followed by four two-monthly injections of aflibercept in the first year. The first-year protocol was identical to that used in the VIEW I/II studies^7,8^. After the first year, treatment was continued at the physicians’ discretion. In most cases, treatment was administered on an as needed basis (pro re nata, PRN).

As previously reported, we investigated optical coherence tomography images at baseline and at year one—when the fixed regimen was finished^11^. Briefly, we measured central retinal thickness, subfoveal choroidal thickness, and the maximum height of the pigment epithelium detachment. The measurement of CRT and subfoveal choroidal thickness was done in horizontal and vertical scans, and the mean value was used for the analysis. The presence of external limiting membrane (ELM)/ellipsoid zone (EZ), vitreoretinal adhesions, and reticular pseudodrusen was also graded by two of the authors (KN and AO). Any discrepancy in grading was arbitrated with a discussion.

We investigated changes in visual acuity as a main outcome measure. Changes in visual acuity in subgroups (who had better baseline visual acuity or fewer number of injections) were also investigated. In addition, we performed multivariate regression analysis to identify the baseline characteristics predicting visual outcome.

### Statistical analysis

The data are shown as mean and 95% confidence interval unless otherwise specified. Statistical analysis was carried out using SPSS version 19 (IBM Japan, Tokyo, Japan). A P-value smaller than 0.05 was considered significant. Visual acuity was measured with Landolt C charts and was converted to logarithm of minimum angle of resolution (logMAR) for the analysis. While we used the results of survivors (who completed four-year follow up), data that included the dropout patients were also analyzed using a *last observation carried forward* policy for sensitivity analysis. To compare changes from baseline, repeated measures ANOVA with post hoc Dunnet correction was applied. Shapiro-Wilk test showed that age followed normal distribution and the other investigated factors followed non-normal distribution. Bivariate relationships were examined using Pearson’s correlation coefficient for age and using Spearman’s correlation coefficient test for the other continuous variables and binary variables. Step-wise multivariate regression analysis was performed with age, CRT, choroidal thickness, presence or absence of ELM, EZ, and vitreoretinal adhesion as independent factors and visual acuity at year four as a dependent factor.

## Results

Among the 98 eyes of 98 patients, 25 (25.5%) dropped out during the follow-up period. Baseline characteristics of survivors and dropouts are shown in Supplementary Table. In summary, dropout patients had worse visual acuity and tended to be male. Reasons for dropout included death (unknown reason, one patient), illness (cerebral infarction, one patient; non-Hodgkin lymphoma; one patient, traffic accident, one patient), and socioeconomic reasons (6 patients). Fifteen patients did not return the hospital for unknown reasons.

**Figure 1:**
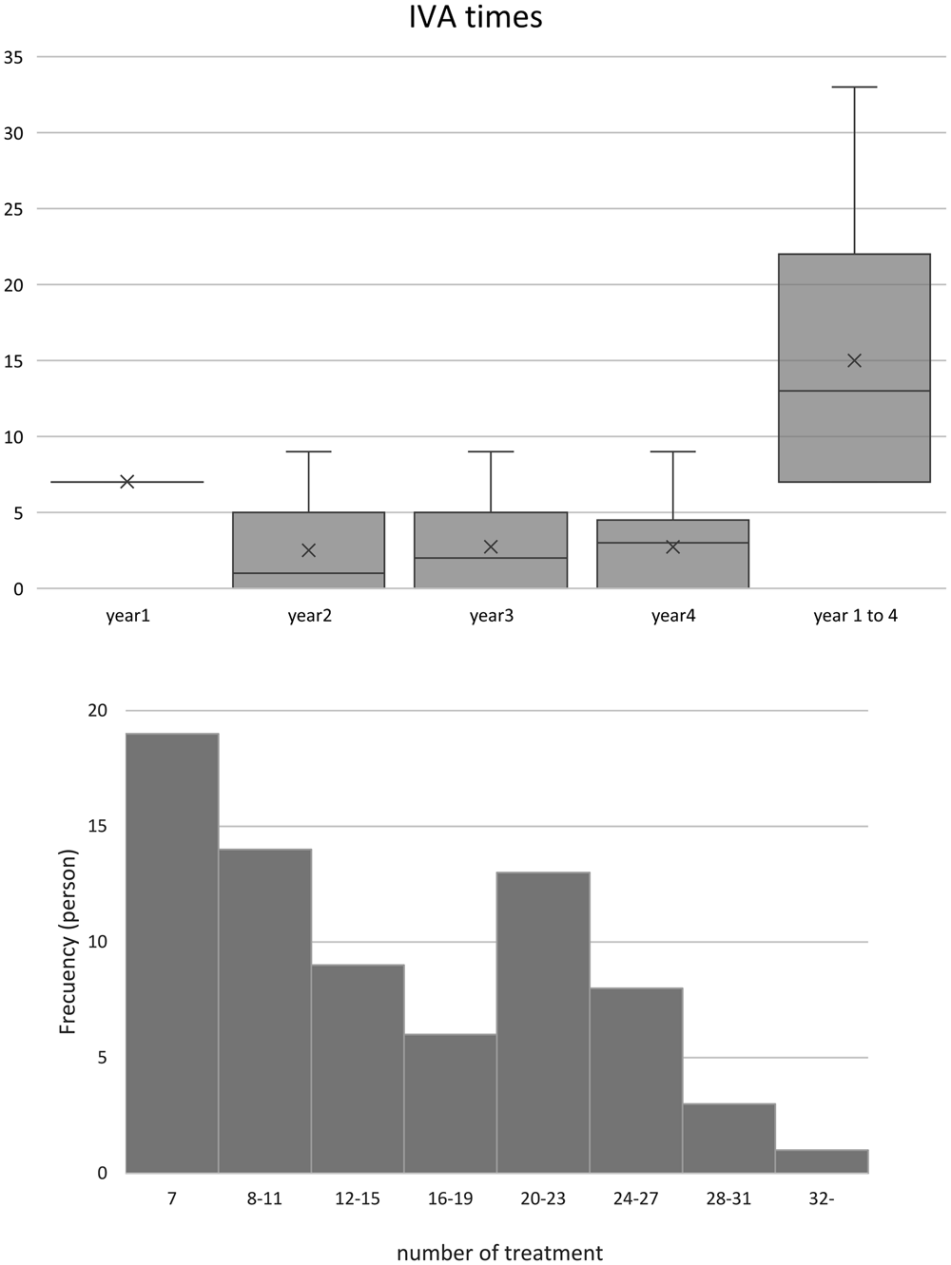
The box and whisker plot shows number of aflibercept injections in eyes with neovascular age-related macular degeneration. (Top) The patients received three monthly injections followed by two-monthly treatments in the first year (seven times). Thereafter, additional treatment was administered at the physicians’ discretion. The mean number of injections was approximately 2.5 in the following years. Frequency distribution of the number of injections is shown in bottom panel. The histogram shows bimodal distribution; some patients required almost no injections in the second, third, and fourth years, whereas others required continuous injections.

The study sample comprised 32 patients with typical AMD, 33 patients with polypoidal choroidal vasculopathy, and 8 patients with retinal angiomatous proliferation. The patients received 7.0 (6.9 to 7.1), 2.5 (1.9 to 3.1), 2.7 (2.1 to 3.4), and 2.7 (2.2 to 3.3) injections at year one, two, three, and four, respectively (Fig. 1; top). Five patients also underwent photodynamic therapy combined with aflibercept. A histogram of the number of injections is shown in Fig. 1 (bottom). The result shows bimodal distribution; some patients required no additional treatment after one year, while others required continuous injections.

**Figure 2:**
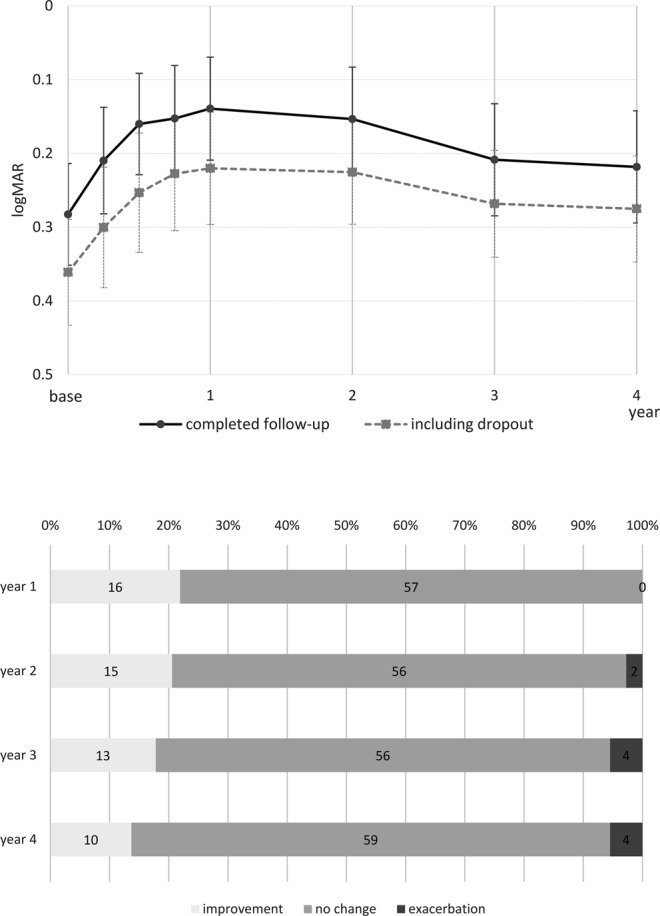
Visual outcome of eyes with neovascular age-related macular degeneration treated with intravitreal aflibercept injections. The solid line in the top panel shows the overall result of eyes, which completed the four-year follow-up. Visual gain in the first year was gradually lost in the following years, but the vision remained above the baseline level even at year four. A dotted line shows the data of all patients including those who dropped out. Missing data were imputed with a *last observation carried forward* policy. The visual course in the cohort was essentially similar despite the difference of baseline visual acuity, indicating the data of survivors can be approximated as a representation of the overall cohort. The bottom graph shows the percentage of patients who gained or lost more than three lines of vision. All the patients gained or maintained vision in the first year. Although some patients lost vision in the following years, 94.5% maintained their vision even at year four.

Visual outcome of patients who completed the four-year follow-up is shown in Fig. 2 (top). Visual gain was 0.14 at year one (0.01 to 0.28, P = 0.033), 0.13 at year two (−0.01 to 0.27, P = 0.069), 0.07 at year three (−0.06 to 0.21, P = 0.557), and 0.06 at year four (−0.07 to 0.20, P = 0.697). For comparison, visual outcome of all patients—including those who dropped out—is shown with a dotted line (Fig. 2 top). Survivors had better baseline visual acuity, but the gain of vision and subsequent loss of vision thereafter was similar with the dropout-imputed data. The prevalence of patients who gained or lost more than 0.3 logMAR is shown in Fig. 2 (bottom). At year one, all the patients had gained or maintained vision. In the following years, some patients lost vision, but 94.5% maintained their vision through year four. Gain of vision was 0.07 (0.01 to 0.14) in typical AMD, 0.08 (−0.01 to 0.16) in PCV, and −0.02 (−0.22 to 0.18) in RAP patients, and there was no difference among the subgroups (P = 0.513).

**Figure 3:**
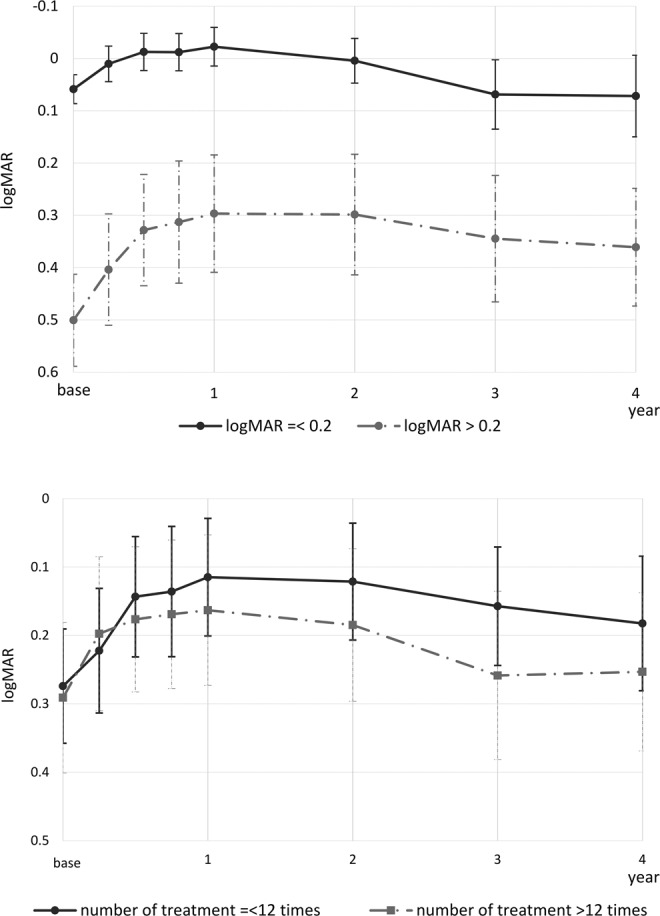
Visual outcome of eyes with neovascular age-related macular degeneration treated with intravitreal aflibercept injections. The top graph shows visual outcome in eyes with a baseline logarithm of minimum angle of resolution (logMAR) better than 0.2, or not. Those with poor visual acuity at baseline gained more vision in the first year. However, both groups showed similar decline of vision in the following year. The visual outcome of the group with poor baseline vision did not reach the same level of visual acuity as the group with good baseline vision. The bottom graph shows visual outcome in eyes that received more or less than 12 injections. Interestingly in this cohort, fewer number of injections was not associated with limited visual gain. Instead, those who received a greater number of injections showed slightly inferior outcomes.

Visual outcome in eyes with baseline logMAR = < 0.2 (36 eyes) and >0.2 (37 eyes) is shown in Fig. 3 (top; cut off value of 0.2 is based on median 0.22). The visual course was different between the cases, as expected (P = 1.5 × 10^−44^). While those with worse baseline logMAR gained greater vision in the first year (0.2 vs 0.08, P = 8.1 × 10^−4^), both groups showed similar changes in the subsequent three years. The change in vision from year one to year four was 0.09 and 0.06, respectively; this difference was not significant (P = 0.552).

We also investigated visual outcome in eyes that underwent less than 12 treatments (36 eyes) and that those that underwent greater than 12 treatments (37 eyes); results are shown in Fig. 3 (bottom; cut off value of 13 is based on the median). Visual gain in the first year was gradually lost in the following years, but the vision remained above the baseline level even at year four in both groups. Two-way ANOVA showed difference in overall visual course between the groups (P = 0.004) despite no significant difference at each time point.

Table 1 shows the result of univariate association between clinical factors at baseline, year one, and visual outcome at year four. The presence of EZ/ELM and choroidal thickness at baseline were associated with final visual outcome. Findings at year one showed a similar tendency; the presence of EZ, ELM, and vitreous adhesion, CRT, and choroidal thickness at year one showed a significant association. After the stepwise variable selection (Table 2), the presence of ELM, vitreous adhesion, and choroidal thickness were found to be significant among baseline factors (Coefficient beta = −0.388, P = 7.34 × 10^−6^; coefficient beta = 0.201, P = 1.31 × 10^−2^; coefficient beta = −7.66 × 10^−4^, P = 2.77 × 10^−2^, respectively), while the presence of EZ, ELM, and vitreous adhesion at year one was associated with visual outcome (Coefficient beta = −0.163, P = 9.87 × 10^−2^; coefficient beta = −0.255, P = 2.79 × 10^−2^; coefficient beta = 0.203, P = 1.85 × 10^−2^, respectively). R^2^ of the model was 0.351 and 0.350, respectively.

**Table 1:** Correlation between clinical characteristics and four-year visual outcome in age-related macular degeneration patients treated with aflibercept.

	Correlation coefficient	*P* Value
**General parameters**		
Age	0.18	0.14
Sex	0.018	0.88
Presence of reticular pseudodrusen	0.23	0.055
**Clinical factors at baseline**		
Central retinal thickness (CRT)	0.10	0.400
Maximum height of the pigment epithelium detachment	0.023	0.850
Choroidal thickness	−0.24	0.038
Presence of EZ	−0.49	1.1 × 10^−5^
Presence of ELM	−0.41	2.7 × 10^−4^
Presence of vitreous adhesion	0.19	0.110
**Clinical factors at year one**		
Central retinal thickness (CRT)	−0.28	0.018
Maximum height of the pigment epithelium detachment	0.23	0.053
Choroidal thickness	−0.25	0.033
Presence of EZ	−0.50	6.8 × 10^−6^
Presence of ELM	−0.47	2.7 × 10^−5^
Presence of vitreous adhesion	0.27	0.023

**Table 2:** Predictors of four-year visual outcome in patients treated with aflibercept, identified with stepwise linear regression analysis.

	Coefficient Beta	95%CI	*P* Value
**Clinical factors at baseline**			
(Constant)	0.648	0.45 to 0.85	3.01 × 10^−8^
Presence of ELM	−0.388	−0.54 to −0.23	7.34 × 10^−6^
Presence of vitreous adhesion	0.201	0.05 to 0.36	1.31 × 10^−2^
Choroidal thickness	−7.66 × 10^−4^	−1.43 × 10^−3^ to −9.92 × 10^−5^	2.77 × 10^−2^
**Clinical factors at year one**			
(Constant)	0.495	0.34 to 0.65	1.33 × 10^−8^
Presence of EZ	−0.163	−0.35 to 0.03	9.87 × 10^−2^
Presence of ELM	−0.255	−0.48 to −0.03	2.79 × 10^−2^
Presence of vitreous adhesion	0.203	0.04 to 0.37	1.85 × 10^−2^

## Discussion

The results of the present study showed that the patients with neovascular AMD, who underwent treatment with aflibercept, maintained vision for four years. Although the maximum visual gain at year one was not retained, 94.5% of patients maintained their vision, which would be an acceptable result in clinical practice. Additionally, we found that the presence of ELM and vitreous adhesion, at baseline or at year one, can be a predictive factor for vision at year four.

The four-year visual outcome in our study was comparable to that of the HORIZON study and the CATT follow-up study. The HORIZON study showed that visual gain decreased to two letters, and maintenance of vision was achieved in 80.4% of patients at year four. The CATT follow-up study reported a loss of three letters and a vision maintenance rate of 87.1% in 5.5 years. The results of the present study showed a four-year visual gain of logMAR 0.06, which corresponds to approximately three letters. The present results confirmed that visual gain in the first year is gradually lost in the following period in real-world clinical practice.

While the visual outcome was similar to that of previous studies, the total number of injections administered in the present study was considerably small. The HORIZON study applied monthly injections during the first two years and then administered them PRN in the following two years. The CATT study cohort examined groups which had received injections monthly, PRN, or with a switch from monthly to PRN, in the first two years. Meanwhile, our study used seven injections in the first year and PRN in the following three years. The mean number of injections in the four years was 27.8 in the HORIZON study, 22.6 (13.3 + 9.3) in the PRN arm of the CATT trial, and 15.0 in our study. We speculated that a longer half-life of aflibercept would partly contribute to the favorable long-term result. Considering that it is not easy to perform continuous anti-VEGF injections in actual clinical practice, fewer additional injections, would be beneficial and ideal.

The visual outcome was largely determined by the baseline vision. Those with worse baseline VA gained greater vision in the first year but the course in the subsequent three years were similar to that of those with good baseline VA. Thus, the gap between the groups were diminished but could never be filled. The result was consistent with previous studies^14,15^. Patients with good visual acuity tended to gain less vision due to the ceiling effect, but they still maintained a higher level of final vision. The result indicates the importance of early detection of the disease and prompt treatment. Although the treatment can be used for those with poor baseline visual acuity, it is difficult to achieve favorable outcomes once the retina is severely impaired.

Fewer number of injections was not associated with poor visual outcome in the present cohort. Considering that AMD is a chronic disease and the anti-VEGF therapy is not a definitive treatment, we expected that cases with fewer injections may lose vision due to undertreatment, as reported in a previous study^16^. However, the present result, together with bimodal distribution of required injections, indicated that a certain percentage of patients were able to retain vision without intensive treatment once the active phase of the disease was controlled. In fact, such remission was also observed with photodynamic therapy^17^ and ranibizumab^18^. While proactive treatment, including fixed and treat-and-extend regimens became the current standard, these protocols may lead to overtreatment in some patients. Identifying good responders may reduce the treatment burden of patients, physicians, and social economy. Individualized treatment protocols with an observation period may be useful to avoid over- and under-treatment^19^. On the other hand, some cases required more than 30 injections in four years. We sometimes applied photodynamic therapy combined with aflibercept (five cases in this cohort) but the result was not consistent. Further studies are needed to establish optimal treatment strategy for the low- or non-responders.

Some of the clinical findings at baseline or at year one were associated with visual outcome at year four. Specifically, EZ and ELM represented good indicator; they reflected photoreceptor integrity, and the association with vision was consistently shown in previous studies^20–22^. Meanwhile, the association of vitreoretinal adhesion was rather unexpected. While the presence of vitreomacular adhesion was associated with neovascular AMD^23^ and the negative effect on visual outcome was reported^24^, the CATT study subanalysis showed no association with visual outcome^25^. Considering the small number of cases with vitreous adhesion (n = 14), the result should be interpreted with some reservation. Thick Choroid at baseline was also associated with better visual outcome as previously reported^26^. Difference in choroidal and choriocapillaris blood supply was suggested as a reason for the finding^26^ but the theory is yet to be proven.

The present study has several limitations including relatively small number of patients and considerable dropout despite that last observation carried forward analysis suggested that dropout did not bias the result critically. Treatment outcome according to CNV subtypes was not significantly different but this can be due to small sample size as well. The finding should be confirmed in larger cohort with multi ethnicities. In addition, the treatment protocol after the first year was at physicians’ discretion. While the result would reflect real-world outcome, more controlled study is needed to confirm scientifically robust evidence of the treatment efficacy.

In conclusion, we showed favorable four-year outcome of aflibercept treatment in patients with neovascular AMD. After one year of a fixed regimen protocol, some patients required fewer injections while others needed continuous injections. The presence of ELM and the absence of vitreous adhesion were positive indicators of vision in the long term. The present result would provide practical information to physicians in this field.

## Supplementary information

supplementary table
