# Supplementary material for: Four-Year Outcome of Aflibercept for Neovascular Age-Related Macular Degeneration and polypoidal choroidal vasculopathy
Source: Sci Rep. 2019 Mar 6;9:3620. doi: 10.1038/s41598-019-39995-5 (PMC6403223; doi:10.1038/s41598-019-39995-5)
Supplement: Supplementary file 1 — supplementary table [file 41598_2019_39995_MOESM1_ESM.docx]

**Four-Year Outcome of Aflibercept for
Neovascular Age-Related Macular Degeneration
and polypoidal choroidal vasculopathy**

Keiichi Nishikawa, Akio Oishi, Masayuki Hata, Masahiro Miyake, Sotaro Ooto, Kenji Yamashiro, Manabu Miyata, Hiroshi Tamura, Naoko Ueda-Arakawa, Ayako Takahashi, Yu Kawashima, Akitaka Tsujikawa

Department of Ophthalmology and Visual Sciences, Kyoto University Graduate School of Medicine, Kyoto, Japan

Corresponding Author: Akio Oishi

Department of Ophthalmology and Visual Sciences, Kyoto University Graduate School of Medicine, 54 Shogoin Kawara-cho, Sakyo-ku, Kyoto 606-8507, Japan

Tel: +81-75-751-3248

Fax: +81-75-752-0933

E-mail: [aquio@kuhp.kyoto-u.ac.jp](mailto:aquio@kuhp.kyoto-u.ac.jp)

Supplementary table

Comparison of survivors and dropouts in patients with age-related macular degeneration treated with aflibercept.

|  |  |  | **Mean (95% CI)** |  |
| --- | --- | --- | --- | --- |
|  |  | **Survivors** | **Dropouts** | ***P* - value** |
| **Variable** | | **(n=73)** | **(n=25)** |  |
| Age, year | | 75.3 (73.6 to 77.0) | 76.8 (73.4 to 80.2) | 0.42 |
|  | < 60 years old (%) | 2.7 | 4.0 |  |
|  | 60-69 years old (%) | 21.9 | 20.0 |  |
|  | 70-79 years old (%) | 45.2 | 24.0 |  |
|  | 80-89 years old (%) | 30.1 | 48.0 |  |
|  | >= 90 years old (%) | 0.0 | 4.0 |  |
| Sex | |  |  |  |
|  | male (%) | 57.5 | 80.0 | 0.056 |
|  | female (%) | 42.5 | 20.0 |  |
| Baseline visual acuity, logMAR | | 0.28 (0.21 to 0.35) | 0.59 (0.42 to 0.76) | 0.003 |
|  | <= 0.2 (%) | 49.3 | 24.0 |  |
|  | > 0.2 (%) | 50.7 | 76.0 |  |
| Central retinal thickness (μm) | | 304 (272 to 336) | 360 (301 to 419) | 0.050 |
| Choroidal thickness (μm) | | 236 (214 to 258) | 279 (234 to 324) | 0.078 |
| Maximum pigment epithelium detachment (μm) | | 248 (225 to 271) | 266 (166 to 366) | 0.725 |
| Presence of vitreous adhesion (%) | | 26.0 | 32.0 | 0.608 |
